# Importance of Hemoglobin SC (HbSC) Screening: Diagnosis of Sickle Cell HbSC Disease Following Hemolytic Crisis and Pulmonary Embolism Due to Peripherally Inserted Central Catheter (PICC Line) Thrombosis

**DOI:** 10.7759/cureus.66628

**Published:** 2024-08-11

**Authors:** Husnain R Ali, Vedangkumar Bhatt, Michael Farina

**Affiliations:** 1 Family Medicine, American University of the Caribbean, New York City, USA; 2 Family Medicine, BronxCare Health System, New York City, USA

**Keywords:** osteomyelitis, general internal medicine, upper extremity dvt, sickle cell complications, hemoglobin sc disease, deep vein thrombosis (dvt), acute pulmonary embolism, sickle cell disease: scd, sickle cell crisis

## Abstract

Sickle cell disease includes various inherited hemoglobinopathies due to the production of abnormal hemoglobin molecules. This can lead to significant clinical complications and sequelae. Hemoglobin SC (HbSC) is a notable variant of SCD, requiring early diagnosis and management to prevent severe outcomes. This case report highlights the critical need for SCD screening, particularly among immigrant populations where current U.S. guidelines do not mandate arrival screening. We present the case of a West African male, age 45, with chronic osteomyelitis, who developed a life-threatening pulmonary embolism (PE) due to peripherally inserted central catheter (PICC line) thrombosis, triggering a hemolytic crisis and thereby revealing HbSC disease. The authors of this report advocate for routine SCD screening in high-risk populations through targeted screening programs. Through multidisciplinary management and public health initiatives, we can address the gap in screening and ensure early detection and treatment of SCD in vulnerable populations.

## Introduction

Sickle cell disease (SCD) encompasses various inherited hemoglobinopathies marked by abnormal hemoglobin molecules, yielding multiple clinical comorbidities. Hemoglobin SC (HbSC) represents a significant variant, requiring early diagnosis for effective management and complication prevention. Our case underscores the importance of SCD and variant screening, especially among immigrant populations lacking established guidelines in the USA. Current guidelines for the immigrant population do not include arrival screening, as it does not pose a public health risk; however, there is a requirement for refugees to be tested with nonspecific tests such as complete blood count with red blood cell indices, white blood cell differential, and platelet count. We present a case where acute-on-chronic osteomyelitis led to a peripherally inserted central catheter (PICC line) insertion for intravenous antibiotics, resulting in a life-threatening pulmonary embolism (PE) due to PICC line thrombosis. This triggered a hemolytic crisis, revealing an undiagnosed HbSC disease. This underscores the need for routine SCD screening, particularly when obscured by concurrent medical conditions. The interplay of acute stressors like PE and osteomyelitis can uncover the hemolytic potential of SCD, leading to severe complications.

According to prevalence data reported by the Agency for Health Care Policy and Research (AHCPR) spanning from 2005 to 2007, of the 104,487 people estimated to have sickle cell disease, 66,070 (63%), including 64,131 African-Americans and 1,946 Hispanics. In another estimate utilizing the U.S.A. Population Census Data, 101,840 African-American individuals were affected along with 2,646 Hispanic individuals [1].

The long-term and short-term complications of sickle-cell disease are extensive, with some of the most common and significant complications to be summarized in brief. Vaso-occlusive episodes, commonly known as pain episodes, are the prototypical symptom of sickle-cell disease. In fact, about half the afflicted population experiences a pain episode; however, the frequency, quantification of severity, and management are still poorly understood and require further research [2]. Anemia, characterized by low hemoglobin levels, is a defining feature of sickle cell disease (SCD), particularly in individuals with homozygous HbS hemoglobin variant (sickle cell anemia, or SCA). It is almost universally present in these patients. Cardiac and pulmonary complications are also significant and notably can include cardiac damage/myocardial infarction, coronary artery ectasia, pulmonary hypertension, acute chest syndrome, and fat-embolism syndrome. A large retrospective study found that cardiac and pulmonary issues accounted for 45% of deaths among patients. Additionally, the occurrence of coronary artery ectasia in patients with SCD was notably higher at 17.7%, compared to only 2.3% in the general population [3]. Fat embolism syndrome had a high mortality rate of 64% in a recent report, which decreased to 29% with prompt exchange transfusion [4]. Priapism occurs in males with a rate of up to 48% and may lead to complications such as erectile dysfunction. Pulmonary hypertension has also been found in studies, with an incidence of 31.8% [5]. Infection also becomes a significant problem due to auto-splenectomy secondary to splenic infarction, leading to vulnerability to encapsulated organisms such as Streptococcus pneumoniae. The rate of mortality from such infections can be between 10% and 30% [6].

While much attention has been focused on the clinical management and screening of sickle cell disease, there is an increasing need to assess sickle cell trait.

It is generally triggered in patients with sickle cell trait by events that affect normal blood circulation such as high altitude, pulmonary embolism, and infections [7]. When red blood cells undergo sickling, they develop a crescent shape and become rigid in structure causing them to block blood vessels and impede circulation [8]. Upon obstructing blood vessels, it leads to premature destruction, also known as hemolysis, causing hemoglobin to freely circulate in the bloodstream. In specific situations, the destruction of red blood cells is too sudden and the body is unable to compensate; this manifests as acute anemia. This is a serious, life-threatening condition and, often, these individuals will require multiple blood transfusions [8].

Furthermore, clinicians must be able to anticipate and resolve many of the complications and sequelae associated with SCD, which often necessitates a multidisciplinary approach in both children and adults. Regular screening and early detection are critical to identify high-risk features and the onset of chronic complications early. Long-term disease-modifying therapies also play a crucial role in decreasing the overall incidence and severity of complications. Management of sickle cell disease is therefore multidisciplinary and is often directed at specific sequelae. First-line therapy is classically hydroxyurea, which reduces the incidence of painful episodes and improves mortality [2]. Vaso-occlusive/pain episodes are treated by analgesics (opioids, nonsteroidal anti-inflammatory drugs (NSAIDs)) and hydration, infection may require antibiotics (prophylactic penicillin in children; acute infection involves coverage against S. pneumoniae and gram-negative enteric organisms) and surgery [9]. Anemia typically requires simple transfusions with the clinician being vigilant to be mindful of aplastic anemia (often secondary to Parvovirus B19), acute chest syndrome, and splenic sequestration crisis. The presence of severe respiratory depression with hypoxemia may require an exchange transfusion instead. Organ-specific damage involving the kidneys may benefit from ACE-inhibitor therapy, cardiac and pulmonary complications may require bronchodilators, and priapism may require surgery. Complications of sickle-cell disease are extensive and require close observation and long-term management [9].

The authors of this article believe that the screening of sickle cell trait is of utmost importance with respect to populations that are often missed or underscreened such as the patient within this case report. This report aims to highlight the prevalence of sickle cell trait among immigrant adults from regions with a high incidence and stress the importance of targeted screening programs. The acknowledgment of this condition in immigrants from certain regions contributes to an inclusive healthcare practice while promoting health equity.

## Case presentation

A 45-year-old West African male with a history of chronic osteomyelitis of the left tibia, developed after a tibial fracture from a motor vehicle accident four years ago. He underwent excisional debridement and a full-thickness skin graft. This was followed by a split-thickness skin graft on the left lower extremity five months later.

The patient arrived at the emergency department with excessive dizziness persisting for three days and severe chest pain for the past two days rated a 10/10 in severity. He also experienced two episodes of syncope and dizziness with hemoptysis. Swelling of the right upper extremity’s PICC line site was seen with the patient endorsing progressive swelling. Further physical exam findings included warmth, erythema, tenderness, and dull pain in the area.

The purpose of the PICC line was for an intravenous antibiotic regimen of vancomycin and meropenem to treat chronic osteomyelitis developed by the patient post motor vehicle collision five years ago.

Along with the PICC line swelling, the emergency department physical exam showed pronounced scleral icterus. The patient then experienced a syncopal episode with tachypnea and febrile. Oxygen saturation decreased to 80%-86%. The placement of a rebreather mask improved O2 saturation to 100%.

**Figure 1:**
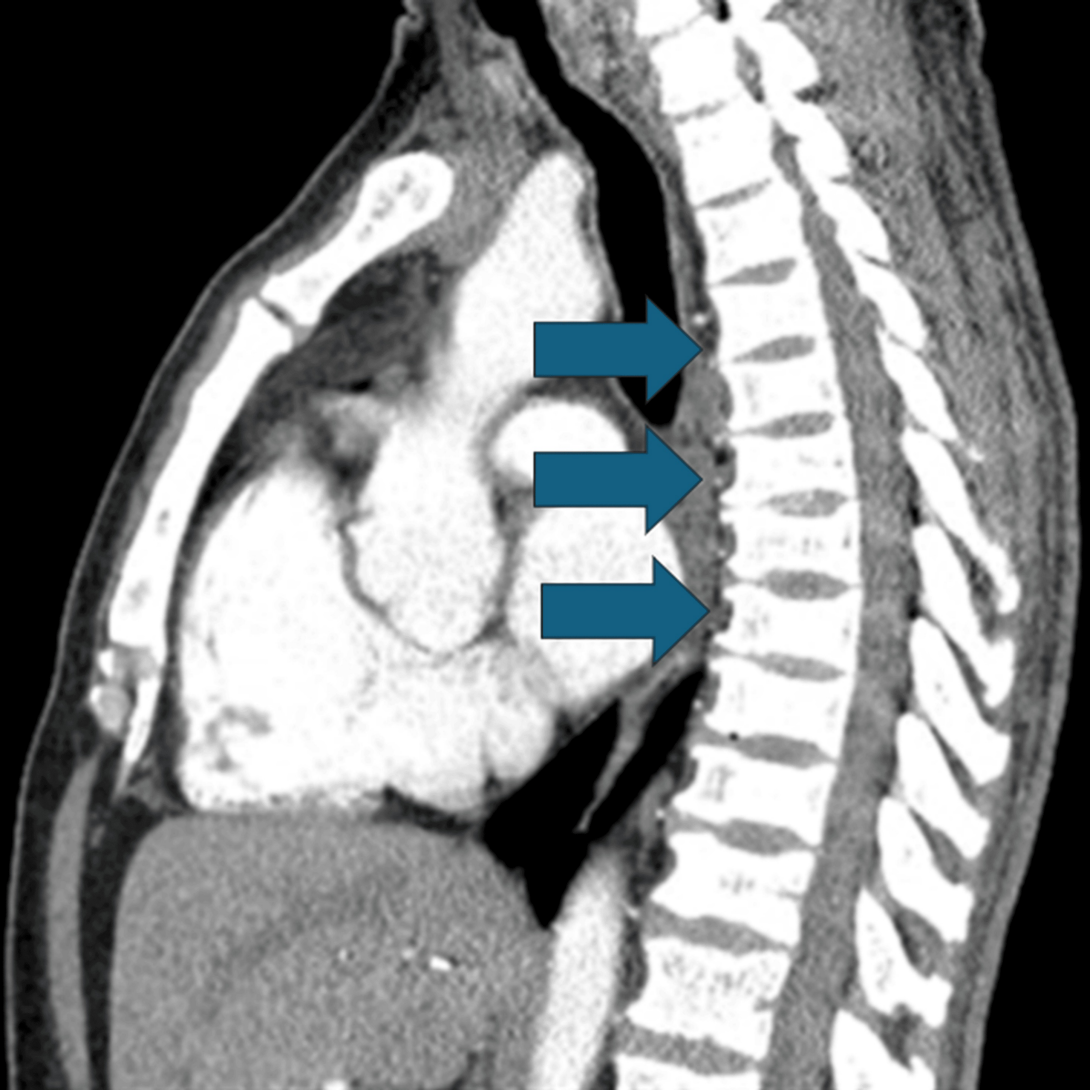
H-shaped vertebrae (Lincoln log vertebrae) were found on presentation with chest CT angiogram and are a finding seen in sickle-cell anemia; blue arrows highlight the affected vertebrae

The patient had a Wells score of 7, thus prompting a chest CT exam (CTA), and D-dimer assay. The CTA revealed bilateral pulmonary embolism and reflux of contrast into the IVC and hepatic veins, indicating right heart dysfunction. The D-dimer was markedly elevated at 2805 ng/mL. Chest X-ray revealed the PICC line in the SVC with no cardiopulmonary disease. The presence of widespread H-shaped vertebral bodies commonly found in the setting of sickle cell trait was also found in the imaging, thus Hematology and Oncology were counseled (Figure 1).

Assessment of pulmonary embolism revealed a minimal likelihood of mortality (Pulmonary Embolism Severity Index (PESI) score of 75). Heparin drip was started and the patient’s blood pressure remained stable (systolic: 150-160 mmHg; diastolic 80-90 mmHg). Pulse ranged from 70-90 bpm and respiratory rate was 16-19 breaths per minute. An echocardiogram revealed an ejection fraction of 60-65% with no wall motion abnormalities.

Interventional radiology consultation determined that the patient was not a viable candidate for thrombectomy. Suspicion of Type 2 non-ST-elevation myocardial infarction (NSTEMI) led to the admission of the patient to the ICU unit (initial troponin T levels: 30 ng/L; repeat troponin T levels: 86 ng/L). ICU course was non-significant with troponin levels downtrending and ECG results being unremarkable.

Heparin administration was ceased due to an initial elevated partial thromboplastin time (PTT) of 270 seconds; however, subsequent confirmatory testing revealed a PTT of 29.2 seconds, indicating that the initial reading was likely inaccurate. Mild thrombocytopenia was noted (114 k/uL) with platelets improving throughout the hospital course (140 k/uL). Heparin was replaced by enoxaparin to optimize anticoagulation. Total bilirubin results were elevated (3.9) but the remaining liver function tests remained within normal limits. A heparin-induced thrombocytopenia (HIT) workup was not pursued due to a low 4T (Thrombocytopenia, Timing of thrombocytopenia, Thrombosis, and oTher reason) score (3). CBC revealed normocytic anemia with haemoglobin at 8.9 g/dL. Ultrasound of the arm for the suspicion of PICC line thrombosis revealed a large thrombus burden in the right subclavian vein. Interventional Radiology removed the PICC line and obtained cultures at that time. The patient was moved to the Family Medicine floor for observation and remained vitally stable but endorsed pain in the right axillary region. Furthermore, the patient’s hemoglobin remained subnormal initially on the Family Medicine hospital course (9.9 g/dL->8.9 g/dL->9.2 g/dL->8.8 g/dL-> 8.2 g/dL) but normalized. Table 1 represents the relevant lab values on presentation to the hospital.

**Table 1:** Relevant lab values on presentation to the hospital

Test Name & Result	Value	Range
Troponin Delta Calculation	56 (HH)	-
Blood Gas with Electrolytes & Creatinine - pH	7.335 (L)	7.350-7.450
Partial Thromboplastin Time (APTT)	270.0	27.2-39.6 second(s)
Prothrombin Time (PT)	12.8	9.9-13.3 second(s)
Complete Blood Count + Automated Diff - WBC Count (White Blood Cell Count)	10.9 (H)	4.8-10.8 k/ul
Complete Blood Count + Automated Diff - RBC Count (Red Blood Cell Count)	3.48 (L)	4.50-5.90 MIL/ul
Complete Blood Count + Automated Diff - HGB (Hemoglobin)	9.9 (L)	12.0-16.0 g/dl
Complete Blood Count + Automated Diff - Hematocrit	29.5 (L)	42.0-51.0 %
Troponin T, Serum - Troponin T, Serum	86	<=12- ng/L
Basic Metabolic Panel - Sodium, Serum	140	135-145 mEq/L
Basic Metabolic Panel - Potassium, Serum	3.8	3.5-5.0 mEq/L
Basic Metabolic Panel - Glucose, Serum	131 (H)	70-120 mg/dL
Activated Partial Thromboplastin Time	138.9	27.2-39.6 second(s)
Magnesium, Serum	Varying	Varying
B-Type Natriuretic Peptide	294 (H)	0-125 pg/mL
D-Dimer Assay, Plasma	2805 (H)	0-230 ng/mL
Respiratory Viral Panel Biofire	COVID-19 Detected	-

PICC line cultures were attempted but were complicated due to inadequate sample volume yielding no growth. Blood cultures returned with Staphylococcus epidermidis, with sensitivities to Amoxicillin/Clavulanate, Ampicillin/Sulbactam, Clindamycin, Erythromycin, Gentamicin, oxacillin, tetracycline, trimethoprim/sulfamethoxazole, and vancomycin. It was determined to continue the treatment of IV vancomycin and meropenem for the remainder of the hospital course.

A hematology consult advised investigating possible hemolysis in the patient. Testing revealed negative direct and indirect Coombs, a negative hepatitis B panel, and a peripheral blood smear, which showed canoe-shaped red blood cells, polychromasia, and target cells. The patient denied any known family history of hematological disorders. Electrophoresis confirmed sickle-cell disease (SCD) SC variant with hemoglobin S and hemoglobin C isoforms (Table 2).

**Table 2:** Hemoglobin electrophoresis results confirming hemoglobin SC disease

Test	Result	Reference Range
Hemoglobin A Subunit (Hgb A)	0.0 L	>96.0 %
Hemoglobin C Subunit (Hgb C)	43.2 H	-
Hemoglobin F Subunit (Hgb F)	4.2 H	<2.0 %
Hemoglobin S Subunit (Hgb S)	49.1 H	-
RBC Count	2.83 L	4.20-5.80 Million/uL
Hemoglobin Level (HGB)	8.1 L	13.2-17.1 g/dl
Hematocrit	23.9 L	38.5-50.0 %
Mean Corpuscular Volume (MCV)	84.5	80.0-100.0 fL
Mean Corpuscular Hemoglobin (MCH)	28.6	27.0-33.0 pg
Red Blood Cell Distribution Width (RDW)	18.6 H	11.0-15.0 %
Hemoglobin A2 Level (HbA2)	3.5 H	2.2-3.2 %

The patient experienced a hemolytic crisis with hemoglobin dropping to 6.9 g/dL, necessitating a transfusion of one unit of packed red blood cells. Due to low suspicion of acute chest syndrome and no evidence of organ failure, exchange transfusion therapy was not pursued. The hematology team also advised against the use of hydroxyurea to preserve the current white blood cell count amid the patient’s bacteremia.

Hemoglobin levels fluctuated between 6.9 and 8.1, with uptrending values to 10.1 at discharge. Along with anemia, pulmonary embolism (PE) and deep vein thrombosis (DVT) were actively monitored. The patient received DVT and PE prophylaxis in the form of apixaban, routine spirometry, physical therapy, and ambulation. Pain and swelling of the arm and pain on inspiration both decreased with the hospital course. Osteomyelitis was monitored via routine complete blood count (CBC), comprehensive metabolic panel (CMP), and C-reactive protein (CRP). Discharge medications included folic acid (for sickle-cell SC) ferrous gluconate (for anemia), ergocalciferol (vitamin D deficiency), apixaban (for DVT), and mupirocin (osteomyelitis). 

## Discussion

The patient in this article with no prior medical history suffered a hemolytic crisis, DVT/PE, and further sequelae, which highlights the need for improved screening and diagnosis of SCD. Two contemporary cases have been selected to illustrate this. A 3-year-old Guinean boy was misdiagnosed with the wrong variant of SCD, leading to a delayed diagnosis. The initial presentation included fever spikes and ankle and wrist joint pain, leading to suspected juvenile idiopathic arthritis, which was treated with NSAIDs. While the fevers stopped, ambulation had worsened, especially on the right tibia. Normocytic anemia was observed with a blood smear showing hypochromia, target cells, poikilocytosis, and sickle cells. Bone scintigraphy revealed a bone abscess, confirming osteomyelitis and thus leading to a true diagnosis of SCD via electrophoresis [10].

A 29-year-old African-American woman was brought into the hospital for a stroke after being found unresponsive. Weeks earlier, she had a fever and anemia, which required a blood transfusion. Thrombocytopenia led to an exchange transfusion for suspected thrombotic thrombocytopenic purpura (TTP). A negative ADAMTS13 result led to discontinuation of the therapy. Initial electrophoresis was interpreted as a beta-thalassemia trait. Despite ongoing severe pain and complications including thrombi, subdural hematomas, and bone degeneration, the diagnosis was then reconfirmed with subsequent electrophoresis to be Sβ+ SCD, leading to a delayed diagnosis [11].

Effective follow-up and PCP involvement are significant in continuous care for SCD, including the monitoring of sequelae, providing vaccinations, and initiating therapies (hydroxyurea) [12]. A survey found that only 20.4% of family physicians felt comfortable managing SCD, with comfort levels varying significantly based on the percentage of African American patients in their practice [13]. As per the U.S. Preventative Taskforce, newborn screening must be performed within two months of birth, allowing for the prompt initiation of follow-up care [14]. In fact, in a randomized control trial, penicillin administration led to a decrease in bacteremia (84% risk reduction) with its daily administration [15].

Specific populations from many countries endemic for SCD have increasing populations who are immigrating to the U.S.A. throughout the years 2010-2017. Western African countries (Nigeria, Ghana, and Cape Verde) experienced increased immigration trends of persons, from 45.2%, 44.15%, and 24%, respectively. Eastern African countries (Ethiopia, Kenya, and Eritrea) saw population increases between 55.7%, 46.8%, and 65%, respectively. Specific estimated cases of immigrants are as follows: 495,625, 158,999, 159,344 (Nigeria, Ghana, and Cape Verde) and 11,308, 126,209, 966 (Ethiopia, Kenya, and Eritrea) [16]. This information, along with the absence of a formal policy on screening immigrants, creates a significant gap in knowledge of carrier status, a factor that can have serious implications on diagnosis and treatment [17]. A 2014 study measured how regions with increased immigration from sub-Saharan Africa to Modena, Italy, exhibited increased rates of being carriers of hemoglobin variant genes. They tested a group of 330 women, 92 learned they were carriers of variant hemoglobin and allowed for the immediate introduction of preventative measures to reduce the possible sequelae of SCD [18].

Certain researchers have suggested that screening in emergency departments (ED) can improve SCD diagnosis. A 1994 study in a dominant African-American hospital found that 4% of black patients tested in the ED had the sickle-cell trait [19]. Another study identified 70 refugees with hemoglobinopathies, including SCD in EDs. The peak in 2016 correlated with an influx from sub-Saharan Africa, with anemia being the main reason for identification. Researchers recommended immediate screening of immigrants [20]. However, limitations included a lack of tailored counseling. Community screening programs in Saint Louis, Missouri, successfully engaged participants at high risk for SCT, with nearly 90% undergoing testing. Notable limitations of participation, in which 60% of people completed both testing and genetic counseling, indicate room for improvement [21].

The factors that reduce screening and cause reduced treatment adherence include poor communication of results between healthcare providers and patients. Recent studies suggest that only 16% of surveyed individuals are informed about their sickle cell trait status, and a mere 37% of parents report receiving direct notification regarding the SCT status of their children [22]. Affordability is another barrier, as this affects the ability to screen persons from low-income settings. Government subsidies, more community screenings, and programs that protect immigration status can help these issues. Finally, patient literacy/understanding of SCD is incomplete or inadequate, further reducing screening. Improved healthcare navigation and patient advocacy can help with these barriers as detailed in Table 3 [23].

**Table 3:** Barriers to access of screening in patients with sickle cell and possible solutions to care

Barrier	Description	Possible Solutions
Cost	Areas with low resources are associated with a decreased ability to screen individuals in the country	Implementation of government subsidies and programs and the use of community screening programs at reduced or no cost and financial assistance
Access to Healthcare Services	Limited access to healthcare due to lack of knowledge of the healthcare system, lack of insurance, and fear of exposure of immigration status	Ensure policies that ensure patient confidentiality while ensuring healthcare status
Navigation Complexities In the Healthcare System	Being able to self-direct oneself through the medical system can be daunting and difficult to access SCD-related services	The use of community health navigators and patient advocacy groups
Cultural and Social Stigma	Cultural beliefs and stigma associated with SCD can deter individuals from seeking screening.	Conduct culturally sensitive community engagement initiatives to educate and reduce stigma around SCD.
Poor Communication of Healthcare Results	Decreased understanding of healthcare results can affect patient adherence to treatments	Enhanced training for healthcare providers and patient access to test results

A new study estimates the annual cost of medical care in the US for people who suffer from SCD exceeds $1.1B [24]. When considering the state of diagnosis and testing before immigration to Western countries, it is important to note that testing has also been improving in significant parts of endemic countries due to improving technologies. Point-of-care testing models (HemoTypeSC: enzyme-linked immunoassay (ELISA)-based point of care test) may aid screening efforts. This technology had an impressive overall sensitivity of 99.5% and a specificity of 99.9% for all hemoglobin phenotypes, including 100% sensitivity and specificity for sickle cell anemia, with costs being less than 2 dollars per test, and did not require complex instrumentation or refrigeration to use [25]. Efficacy has been proven in countries such as Nigeria, which conducted a study that found that in the screening of 313 newborn babies, there was a complete concordance of the point-of-care testing of HemoTypeSC to the gold-standard high-performance liquid chromatography (HPLC), boasting a sensitivity and specificity of 100% [26].

Being able to further address the need for effective screening for chronic disease relies on inter-medical collaboration. One proposed model that can be beneficial to these patients includes the chronic care model (CCM), which has shown promise in a systematic review comprising 16 studies. Implementing the CCM was linked to improved diabetes management, attributed to organizational changes that prioritize leadership, utilize Disease Registries, and incorporate electronic medical record systems (EMRS). These changes emphasize patient-centered goals, enable progress monitoring, and crucially, identify gaps in care [27].

## Conclusions

In conclusion, this case report describes the importance and immediate need to address sickle cell trait screening in immigrant populations. The potential for hemolytic crisis in patients with the sickle cell trait is by itself a significant reason to reevaluate screening methods in the United States of America. Current protocols for screening immigrant populations arriving in the United States of America are lacking when it comes to the sickle cell trait. With ever-increasing global migration, diverse communities with varying geographic origins must develop effective screening tools. By working collectively through public health campaigns and physician education programs, we can reduce health disparities within immigrant communities.
